# Viral Load in COVID-19 Patients: Implications for Prognosis and Vaccine Efficacy in the Context of Emerging SARS-CoV-2 Variants

**DOI:** 10.3389/fmed.2021.836826

**Published:** 2022-01-31

**Authors:** Severino Jefferson Ribeiro da Silva, Suelen Cristina de Lima, Ronaldo Celerino da Silva, Alain Kohl, Lindomar Pena

**Affiliations:** ^1^Laboratory of Virology and Experimental Therapy (LAVITE), Department of Virology, Aggeu Magalhães Institute (IAM), Oswaldo Cruz Foundation (Fiocruz), Recife, Brazil; ^2^Leslie Dan Faculty of Pharmacy, University of Toronto, Toronto, ON, Canada; ^3^MRC-University of Glasgow Centre for Virus Research, Glasgow, United Kingdom

**Keywords:** SARS-CoV-2, prognosis, disease severity, emerging variants, vaccines, transmissibility, lethality, viral load

## Abstract

The worldwide spread of the severe acute respiratory syndrome coronavirus 2 (SARS-CoV-2) has caused an unprecedented public health crisis in the 21st century. As the pandemic evolves, the emergence of SARS-CoV-2 has been characterized by the emergence of new variants of concern (VOCs), which resulted in a catastrophic impact on SARS-CoV-2 infection. In light of this, research groups around the world are unraveling key aspects of the associated illness, coronavirus disease 2019 (COVID-19). A cumulative body of data has indicated that the SARS-CoV-2 viral load may be a determinant of the COVID-19 severity. Here we summarize the main characteristics of the emerging variants of SARS-CoV-2, discussing their impact on viral transmissibility, viral load, disease severity, vaccine breakthrough, and lethality among COVID-19 patients. We also provide a rundown of the rapidly expanding scientific evidence from clinical studies and animal models that indicate how viral load could be linked to COVID-19 prognosis and vaccine efficacy among vaccinated individuals, highlighting the differences compared to unvaccinated individuals.

## Introduction

The emergence of coronavirus disease 2019 (COVID-19), caused by severe acute respiratory syndrome coronavirus 2 (SARS-CoV-2) has resulted in a worldwide emergency through rapid expansion of the virus (1). Clinically, most COVID-19 patients present mild or moderate symptoms, but ~15% of infected patients progress to pneumonia and 5% eventually develop more critical manifestations including acute respiratory distress syndrome (ARDS), and multiple organ dysfunction or failure (2, 3). Many studies have sought to elucidate predictors for COVID-19 severity in order to guide clinical management and prognosis of the disease and shed light into new therapeutic strategies (4). With this in mind, a growing body of evidence suggests that severe forms COVID-19 are associated with pronounced lymphopenia, lymphocyte dysfunction and activation, monocyte and granulocyte abnormalities, cytokine storm (increased levels of IL-1β, IL-6, IL-2, IL-8, IL-17, IP10, MCP1, MIP1α, G-CSF, GM-CSF, and TNF-α), high levels of C-reactive protein (CRP), D-dimer, immunoglobulin G (IgG), and total antibodies (4–7). Additionally, a number of reports have investigated the correlation between high viral loads and COVID-19 severity, where the results demonstrated high, little, or no statistical correlation with COVID-19 disease severity (6, 8–19).

As of December 28, 2021, SARS-CoV-2 accounted for more than 281.6 million infections and over 5.4 million deaths across the world-wide human population (1). Approximately 2 years have passed since the emergence of the virus, SARS-CoV-2 genomes are being routinely monitored through epidemiological investigations, virus genetic sequence-based surveillance, and shared at an unprecedented rate, with more than 6.5 million SARS-CoV-2 sequences available via the Global Initiative on Sharing All Influenza Data (GISAID), permitting near real-time surveillance and track the emergence of SARS-COV-2 mutations and new variants (20). Although most mutations in the SARS-CoV-2 genome are expected to be either neutral or deleterious, a proportion of these mutations will affect functional and viral properties in a way that confers a fitness advantage, which may alter infectivity, tropism, virulence, transmissibility, and/or interactions with host immunity (21). Beneficial mutations tend to occur in the minority of cases, when compared to negative effect or no effect “neutral” mutations (21). Notably, many of these beneficial mutations are due to non-synonymous nucleotide substitutions in key areas of the immunodominant spike protein of SARS-CoV-2, resulting in a change in amino acid. Five main variants of concern (VOCs) have emerged since the beginning of the pandemic and have attracted the most widespread attention: alpha (B.1.1.7) in the United Kingdom, beta (B.1.351) in South Africa, gamma (P.1) in Brazil, delta (B.1.617.2) in India, and omicron (B.1.1.529) in Botswana and South Africa (22–27). These variants have been associated with increased transmissibility, viral load, disease severity, evasion of immunity from infection and vaccinations, and reduced susceptibility to monoclonal antibody therapies (22, 23, 28–30), resulting in a catastrophic impact on SARS-COV-2 infection.

**Figure 1:**
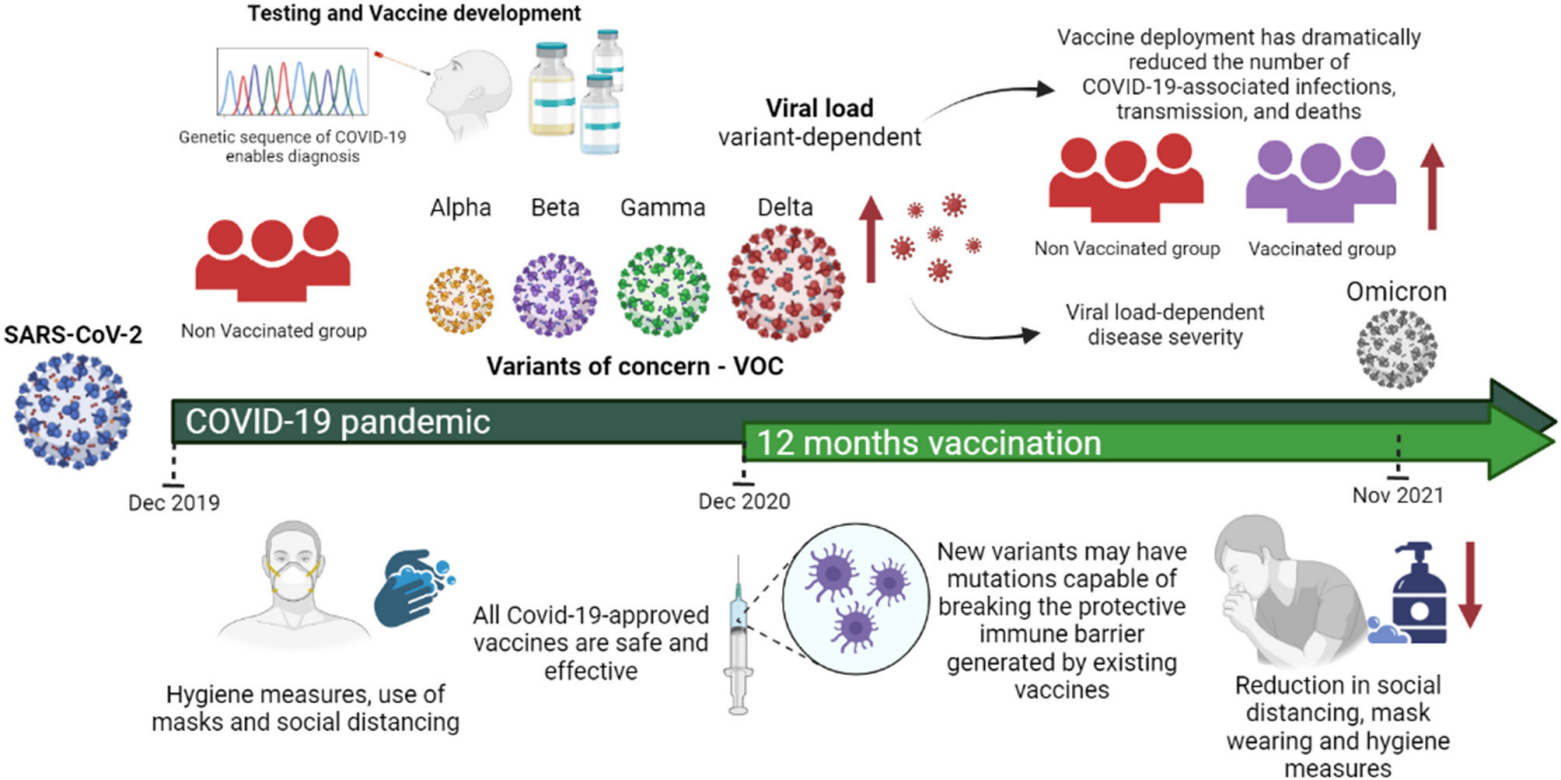
SARS-CoV-2 variants of concern and their impact on viral load, disease severity and vaccine efficacy. All approved COVID-19 vaccines are effective against the variants in circulation. However, in general the effectiveness is slightly lower against them and new variants may have mutations capable of breaking the protective immune barrier generated by existing vaccines. Vaccines together with mitigating measures such as social distance, mask wearing, ventilation and hygiene are important to counter the spread of these variants. Evidence from clinical studies and animal models has indicated that SARS-CoV-2 infectious dose is one likely determinant of ultimate COVID-19 severity and prognosis. However, reduction in hygiene care, mask wearing and social isolation further greater viral transmissibility. In summary, most studies have concluded that vaccination reduces the risk of infection by VOC infection and accelerated viral clearance. Figure 1 was created with Biorender.com under academic license.

Based on the scientific knowledge published so far, we summarize the main characteristics of the emerging variants of SARS-CoV-2, including their impact on viral transmissibility, viral load, disease severity, vaccine breakthrough, and lethality among COVID-19 patients. We also discuss the rapidly expanding scientific evidence from clinical studies and animal models that indicate how viral load could be linked to COVID-19 prognosis and vaccine efficacy among vaccinated individuals, shedding light the differences compared to unvaccinated individuals (Figure 1).

## Emerging SARS-CoV-2 Variants of Concern

During replication of SARS-CoV-2, the RNA-dependent RNA polymerase introduces mutations in the viral genome which may be subjected to selection pressures and then fixed in the population. It has been estimated that an average of 0.5 mutations are accumulated in every person during infection cycle (31). Given the high transmission rates of SARS-CoV-2 around the world, virtually every single base mutation is being generated *de novo* and transmitted daily to a new human host (31). To give rise to new variants, a SARS-CoV-2 mutant must overcome selection pressures and successfully establish a transmission chain among humans (31), which is the major bottleneck for SARS-CoV-2 inter-host dynamics, in which most mutated viruses do not transmit from their original host to another person (32). But despite this, several SARS-CoV-2 variants have been emerging and circulating widely since the beginning of the COVID-19 pandemic (Table 1).

**Table 1:** Characterization of SARS-CoV-2 variants of concern.

**SARS-CoV-2 variant**	**Country emergence/first detection**	Number of countries affected/world cumulative prevalence*	**Spike mutations**		**Impact**				
				**Viral load**	**Transmissibility**	**hACE2 binding**	**Disease severity**	**Immune escape**	**Vaccine efficacy**
Alpha (B.1.1.7)	United Kingdom/September, 2020	169 | 21%	Δ69–70 del, Δ144 del, N501Y, A570D, D614G, P681H, T716I, S982A, D1118H	High (33–37)	50–70% Higher (33, 38, 39)	2–5-fold higher (40)	Higher disease severity (41) Higher Lethality (~60%) (42) Increased severity (hospitalization and mortality (43) Negligible risk of reinfection (44)	Minimal reduction in neutralization (40) Low resistant to monoclonal Abs (45) Modest to reduction in the neutralization and efficacy of sera from convalescent patients or vaccine (36, 41, 46) Minimal impact on neutralization by convalescent and vaccine sera (47–50)	BNT162b2 (Pfizer): 89.5–93% (40, 51, 52) AZD1222 (AstraZeneca): 75–84% (40, 49) NVX-CoV2373 (Novavax): 85.6–96% (40, 53, 54) Ad26.COV2.S (Johnson & Johnson): 70–72% (55)
Beta (B.1.351)	South Africa/October, 2020	117 | 1%	D80A, D215G, L241del, L242del, A243del, K417N, E484K, N501Y, D614G, A701V	2.5 less than delta variant (56)	20–113% higher (33, 40, 41, 57, 58)	5-fold higher (40, 59)	High reinfection rates (41) Higher ratio of hospitalization (60) Possible increase risk of in-hospital mortality (61)	6–7 fold reduced neutralization of human convalescent plasma and RBD, NTD targeting mAbs (40, 43) Elevate resistance to Abs than alpha variant (62) Significant reduction in the neutralization efficacy of sera from convalescent patients or vaccines recipients (41, 62, 63)	mRNA-1273 (Moderna): 96.4% (64) BNT162b2 (Pfizer): 75% (40, 52) AZD1222 (AstraZeneca): 10–81.5% (40, 49, 65) Ad26.COV2.S (Johnson & Johnson): 57–72% (40, 55) NVX-CoV2373 (Novavax): 49–60% (54)
Gamma (P.1)	Brazil/ November 2020	86 | 2%	L18F, T20N, P26S, D138Y, R190S, K417T/N, E484K, N501Y, D614G, H655Y, T1027I, V1176F	Low CT values (24, 28)	~161% higher (33, 41, 66)	2–3-fold higher (40, 67)	High reinfection rates (41) Possible increased risk of hospitalization (60) Increased lethality (~80%) (42)	Reduced neutralization by human convalescent plasma (68) Resistant to neutralizing Abs (28, 60, 69) Significant reduction in the neutralization efficacy of sera from convalescent patients or vaccine recipients (40–42, 46)	mRNA vaccines: 77% (70) AZD1222 (AstraZeneca): 64.1–70.4% (71) Ad26.COV2.S (Johnson & Johnson): 68% (55) CoronaVac (Sinovac): 50.4% symptomatic and 78% mild SARS-CoV-2 Infection (72)
Delta (B.1.617.2)	India October, 2020	171 | 54%	T19R, V70F, G142D, E156del, F157del, R158G, A222V, W258L, K417N, L452R, T478K, D614G, P681R, D950N	High (73)	50–60% more transmissible than alpha (40, 74, 75)	High affinity (76, 77)	Secondary household attack rate elevated (78) Increase of hospitalization risk (79) Increased oxygen requirement, ICU admissions and deaths (80)	3–8-fold reduction in neutralization by vaccine sera and human convalescent plasma (81) Increase of resistance to monoclonal Abs (82)	BNT162b2 (Pfizer): 75–88% (51, 81) AZD1222 (AstraZeneca): 53–67% (40, 51, 83) Bharat Biotech: 65% (40)
Omicron (B.1.1.529)	Botswana and South Africa November, 2021	41 | <0.5%	A67V, del69/70, T95I, G142D, del143/145, L212I, Ins214EPE G339D, S371L, S373P, S375F, Q493R, G496S, Q498R, N501Y, Y505H, T547K, D614G, H655Y, N679K, P681H, D796Y, N856K, Q954H, N969K, L981F, K417N, N440K, G446S, S477N, T478K, E484A, N764K	Possible high (84–86)	10-fold more infectious than original virus or about twice as infectious as the delta VOC (*in silico*) (84, 87)	Strengthens the affinity (*in silico*) (87)	Increase COVID-19 severity (?) Increase hospitalization and death (?) Increase in case of reinfection in South Africa (86)	Reduce neutralization by most of a large panel of potent monoclonal antibodies and antibodies under commercial development (88) Vaccine-escape capability is about twice as high as that of delta (*in silico*) (87) May compromise mAbs and reduce the efficacy of antibodies (*in silico*) (87)	Decrease efficacy (88–90)

Given the evolution of SARS-CoV-2 as the pandemic continues, a US government interagency group has developed a variant classification scheme to categorize mutant viruses that have arisen in the human population. The variants of concern (VOC) are the most relevant. According to their definition, a VOC is “a variant for which there is evidence of an increase in transmissibility, more severe disease (for example, increased hospitalizations or deaths), significant reduction in neutralization by antibodies generated during previous infection or vaccination, reduced effectiveness of treatments or vaccines, or diagnostic detection failures” (27).

The first SARS-CoV-2 VOC was the alpha variant, which emerged in September 2020 in the UK and rapidly become the dominant circulating variant spreading for 169 countries, presenting a cumulative world prevalence of 21% (https://outbreak.info/situation-reports). This variant belongs to Pango lineage B1.1.7, derived from clade 20I (V1) (91). The spike protein harbors several most mutations (Δ69–70 deletion, Δ144 deletion, N501Y, A570D, D614G, P681H, T716I, S982A, D1118H) (33, 92). Phylogenetic analyses indicate that the alpha variant was associated with a growth rate estimated to be 40–70% higher than that of other SARS-CoV-2 variants (38). The N501Y and A570D mutations, located in the region binding domain (RBD), have been related to increase of viral binding affinity to ACE2 host receptor, contributing to transmissibility (33, 57, 93). Others such as P681H, exclusive of alpha VOC and localized adjacent to the furin cleavage site, may be involved in membrane fusion and immune escape (66). The Δ69–70 and Δ144 deletions may to affect the viral recognition, by neutralizing antibodies (Nabs), the testing kit failures (94, 95) and antibody escape (96). Previous studies in hamsters infected by SARS-CoV-2 and human for N501Y, Δ69 deletion and Δ70 deletion, observed a relation between these mutations with high viral load levels in nasal secretions and upper airway (in hamsters), and human airway epithelial cells (97). As well as the ancestor B.1 strain, the alpha variant could produce a high viral load, leading to a low Ct value in RT-qPCR-based diagnosis (47) while being ~50% more transmissible (33, 38, 39, 98). Previous analysis by Public Health England suggested an increase of death risk in patients infected by alpha VOC (99–101). The reinfection risk is very low (39). Mutations in alpha VOC have been related to reduce the neutralizing activity of monoclonal antibody-based therapies. Alpha VOC is refractory to neutralization by most monoclonal antibodies (mAbs) targeting the N-terminal domain (NTD) and is relatively resistant to mAbs against the RBD (102, 103). It is susceptible to neutralizing mAbs as well as by most plasma samples from previously infected and vaccinated individuals (29, 47, 63, 104–107). The sera from individuals who received the BNT162b2 (Pfizer) vaccine or convalescent sera of individuals who recovered from COVID-19 reduced neutralizing activities (47). Some studies have shown a 3-fold to 10-fold reduced susceptibility to 15% of plasma samples from recipients of an authorized mRNA vaccine (47, 104–106, 108, 109). Individuals vaccinated with mRNA vaccines [BNT162b2 (Pfizer) and mRNA-1273 (Moderna)] and adenovirus-vector (Ad26.COV2.S – Johnson & Johnson) may significantly neutralized the alpha VOC compared to the D614G variant (110). The BNT162b2 (Pfizer) vaccine demonstrated an effectiveness of 48.7% (CI, 45.5–51.7%) after one dose, while it was 93.7 (91.6–95.3%) after two doses among infected individuals with the alpha VOC (51). With the AZD1222 (AstraZeneca) vaccine, the effectiveness after one dose was similar to the results found with the BNT162b2 (Pfizer) vaccine, while it was 74.5% (95% CI, 68.4–79.4) after two doses among infected persons with the alpha VOC (51). For mRNA-1273 (Moderna) vaccine against RT-qPCR positive infections, the effectiveness was 88.1% (CI, 83.7–91.5%) and 100% (CI, 91.8–100%) after the first and second doses, respectively (64). Other clinical trials with the BNT162b2 (Pfizer) vaccine, conducted in Israel and Qatar, showed an efficacy > 90% against alpha variant (52, 111). When compared to NVX-CoV2373 clinical trial, the vaccine efficacy was 86.3% against alpha variant compared with 96.4% against non-alpha variants (53).

In October 2020, in South Africa, researchers found a new SARS-COV-2 B.1.351 variant, derived from the 20H (V2) clade and named beta variant by WHO (92, 93). Currently, this variant is present in more than 117 countries and has an accumulated worldwide prevalence of around 1% (https://outbreak.info/situation-reports). The beta variant has ten mutations (D80A, D215G, L241del, L242del, A243del, K417N, E484K, N501Y, D614G, and A701V) in the spike protein. Two new mutations (E484K and K417N) were identified in RBD region (25). The alpha and beta VOCs share two mutations (N501Y and D614G). Notably, these mutations in RBD region (N501Y, E484K, and K417N) may to enhance the binding affinity to human ACE2 receptor (33, 57), which can contribute as a critical role in the SARS-COV-2 transmission (58, 112). Mathematical modeling studies indicated that the beta variant is around 50% more transmissible than pre-existing SARS-CoV-2 variants (113). Reinfections have been reported, indicating immune evasion (114). Little is known whether the beta variant is associated with higher viral levels or disease severity, because once detected it was no longer co-circulating with other SARS-CoV-2 variants. In many Sub-Sahara Africa countries, the beta variant was responsible for more than 50% of infections (https://nextstrain.org/sars-cov-2/). In K18-hACE2 transgenic mice, alpha and beta VOCs induced pathogenic patterns and were 100-fold more lethal than early SARS-CoV-2 lineages (115). The European Centre for Disease Prevention and Control (ECDC), compared the COVID-19 severity cases between VOCs and non-VOCs, and observed that the beta variant was associated with a higher ratio for hospitalization (3.6) (60). The intensive care unit (ICU) and death risk data showed no difference in relation SARS-CoV-2 variants (60). The beta variant has been associated with reduced susceptibility to many mAbs and neutralizing Abs even in vaccinated or previously infected individuals, due to the E484K and K417N mutations (59, 60, 62). A differential susceptibility to neutralizing activities in beta VOC convalescent individuals has been observed. Indeed 46% of convalescent plasma samples displayed 3-fold to 10-fold reduced susceptibility, and 22% of these, >10-fold reduced susceptibility in comparison to early SARS-CoV-2 variants (29, 48, 59, 62, 106, 107, 109, 116–120). Vaccine neutralizing activities also have been evaluated. Individuals that received one of the mRNA vaccines [BNT162b2 (Pfizer) or mRNA-1273 (Moderna)] showed 3-fold to 10-fold (45% of plasma samples) and >10-fold (30% of plasma samples) reduced beta variant neutralizing activity (29, 46, 63, 69, 102, 107, 120–122). In relation to the adenovirus-based AZD1222 (AstraZeneca) vaccine, 42% of plasma samples had 3-fold to 10-fold, and 54% had >10-fold reduced beta variant neutralizing activity (81, 120, 123). With regard the efficacy of vaccines, a study carried out in Qatar-based individuals, revealed that the mRNA-1273 (Moderna) had an efficacy of 61.3 and 96.4% after the first and second doses against the beta variant, respectively (64). On the other hand, the NVX-CoV2373 (Novavax) vaccine showed an efficacy of 60% against the beta variant (54).

The gamma variant was first detected in four travelers returning to Japan from Amazonas state of Brazil in January 2021, but its emergence occurred in November 2020 (24, 28, 124). Currently, the gamma VOC is spread across 86 countries, with an accumulated worldwide prevalence of 2% (https://outbreak.info/situation-reports). The gamma variant has 12 missense mutations (L18F, T20N, P26S, D138Y, R190S, D614G, H655Y, T1027I, V1176F, K417T, E484K, and N501Y) in the spike protein, three of these (N501Y K417N and E484K) are located in the receptor-binding domain (RBD) (28). The triplet of K417T, E484K, and N501Y have been associated to increase virus binding affinity to human ACE2 receptor, which may contribute to increased transmissibility (28, 125). The gamma variant may be 1.7- to 2.4-fold more transmissible that previous SARS-CoV-2 variants (28). The emergence of this variant was associated with a resurgence of COVID-19 in Manaus, Brazil, resulting in an abrupt increase in the number of cases and deaths in this part of the world in January 2021 (22, 24, 28, 126). At the same time, the gamma variant was estimated to result in virus levels 3–4 times higher than earlier SARS-CoV-2 variants, being responsible for an estimated 1.1-fold to 1.8-fold higher mortality (28). Other studies reported a high proportion of COVID-19 infections in several South American and Caribbean countries and about 10% of USA infection rates in June 2021 (127). Additionally, a related study showed an increased risk for hospitalizations and ICU admission, comparing the disease severities among VOCs (B.1.1.7/SGTF, B.1.351 and P.1) and non-VOCs (60). Some mutations, such as L18F, may interfere in the binding of the NTD of spike-targeting neutralizing Abs (128). The resistance profile to the gamma to FDA EUA-approved mAbs is comparable to that of the beta variant (43, 129–131). Antibodies produced by natural infection or vaccines may be less likely to neutralize the gamma (69, 132). SARS-CoV-2 with the E484K might escape neutralization by Abs from convalescent plasma of recovered individual infected with earlier SARS-CoV-2 strains (74). A nationwide case-control study evaluating the protection at 7 days after the second dose of mRNA vaccine against the VOCs (alpha, beta, and gamma) and other non-SARS-CoV-2 variants in France estimated an effectiveness of 88% (alpha), 86% (beta) and 77% (gamma) against COVID-19 (70).

The fourth VOC, called delta by WHO, emerged in October 2020 in India (133) and it has spread to over 171 countries, becoming dominant with an accumulated worldwide prevalence of 54% (https://outbreak.info/situation-reports). Belonging to B.1.617.2 Pango lineage, derived from the 21A clade, the delta variant has 14 mutations (T19R, V70F, G142D, E156del, F157del, R158G, A222V, W258L, K417N, L452R, T478K, D614G, P681R, D950N) in the spike protein, among which only D614G is common to previous circulating SARS-CoV-2 variants (92). The spike RBD region carries 2 new non-synonymous mutations (L452R and T478K) and a deletion (del157). The L452R mutation may stabilize the interaction between the spike and the ACE2 receptor, increasing infectivity (74, 76, 134). P681, located near the furin cleavage site, may to optimize spike cleavage, which in turn may impact on transmissibility. Some studies have revealed that this variant has about 50% higher transmissibility compared to alpha variant (75, 92, 135). Despite differences between countries, Campbell et al. estimated a change in effective reproduction number of SARS-CoV-2 variants in 64 countries (data until 3 June 2021) (30). That study estimated the increase in transmissibility of VOC relative to non-VOC: alpha-29% (95% CI: 24–33), beta-25% (95% CI: 20–30), gamma-38% (95% CI: 29–48), and delta-97% (95% CI: 76–117) (30). In addition to enhanced transmissibility, individuals infected with the delta variant have higher viral load and sheds virus for longer periods (73), impacting the severity disease. The comparison between the virulence of the delta VOC and the non-VOC revealed a remarkable risk for disease severity associated to delta variant, with the increased hospitalizations, higher oxygen requirement, ICU admissions, and deaths (80). An *in vitro* study revealed that the delta variant (containing the mutations G124D) was 6-fold and 8-fold less sensitive to serum neutralizing Abs from recovered persons and vaccine-elicited Abs, respectively, compared to wild type D614G containing SARS-CoV-2 (77, 136). The delta variant may be resistant to neutralization by some anti-N-terminal domains and anti-RBD mAbs, including bamlanivimab (81). The presence of L452R in RBD decreased recognition by mAbs (76, 82, 137). The convalescent sera of individuals up to 12 months after the onset of symptoms were 4-fold less potent against the delta variant compared to alpha variant (81). Similarly, plasma samples obtained from recipients of BNT162b2 (Pfizer) and AZD1222 (AstraZeneca) vaccines displayed a reduction of neutralizing activity against the delta variant (65, 81, 121, 138). Even in the absence of the spike mutations, N501Y and E484K, the delta VOC was found to spread faster in the body and *in vitro* studies reveal lesser sensitivity to the BNT162b2 (Pfizer) vaccine (47, 121). Other studies have demonstrated that the BNT162b2 (Pfizer) and AZD1222 (AstraZeneca) vaccines were 85 and 60% effective against delta variant in the UK (83, 139). In the UK, previous studies have demonstrated that the effectiveness after the first dose of BNT162b2 (Pfizer) and AZD1222 (AstraZeneca) vaccines was 35.6 and 30.0% against symptomatic disease by delta variant. Following the second dose, the effectiveness was 88 and 67% for BNT162b2 (Pfizer) and AZD1222 (AstraZeneca) vaccines, respectively (51). In relation to COVID-19 hospitalization by alpha and delta VOCs vaccinated (mRNA-1273 or AZD1222), a Scottish study cohort observed among delta VOC individuals a strong vaccine effect in reducing the risk of hospital admission compared to unvaccinated individuals (83). The BNT162b2 (Pfizer) vaccine, at least 14 days after the second dose, offered 92 and 79% of protection against alpha and delta VOC infections, respectively (83), while the AZD1222 (AstraZeneca) vaccine offered 73 and 60% of protection against alpha and delta VOCs, respectively (83).

The fifth SARS-CoV-2 variant that has emerged so far is the omicron (B.1.1.529), which was first reported in Botswana and South Africa, November 2021 (26). This variant was detected on 6 continents within a month of its initial discovery and has raised concerns around the world. The omicron VOC is a heavily mutated SARS-CoV-2, with 30 amino acid substitutions, deletion of six residues, and insertion of three residues in the spike protein, mostly concentrated around the receptor binding motif (88). Among the mutations, an insertion (ins214EPE) in spike that was not been previously observed in other SARS-CoV-2 variants (140). It has been hypothesized that this insertion could have been acquired by template switching involving the genome of a low pathogenic coronavirus which can cause the common cold: HCoV-229R (140). A recent study has been estimated that the omicron variant is least ten times more infectious than the wild-SARS-CoV-2 and about twice as infectious as the delta variant (87). Analyzing the replication competence and cellular tropism of the wild-type virus, D614G, alpha, beta, delta and omicron variants in *ex vivo* explant cultures of human bronchus and lung, it was found that the omicron variant replicated faster than all other SARS-CoV-2 variants in the bronchus but less efficiently in the lung parenchyma (141). Based on *in silico* studies, it has been estimated that omicron may be twice more likely to escape immunity generated by current vaccines in comparison to the delta variant (87). It has been demonstrated that the omicron variant was associated with a substantial decrease in neutralization titer of vaccinated individuals with two doses [BNT162b2 (Pfizer) and AZD1222 (AstraZeneca)] (89). Similarly, by using the sera from 25 BNT162b2 (Pfizer) and 25 Coronavac (Sinovac) vaccine recipients, it was found that only 20% of BNT162b2 recipients had detectable neutralizing antibody against the omicron variant, while none of the Coronavac recipients had detectable neutralizing antibody titer against the Omicron VOC (90). These findings suggest that the omicron variant may be associated with lower COVID-19 vaccine effectiveness. Regarding the effect of neutralizing activity against monoclonal antibody-based therapies, recent findings showed that the omicron VOC substantially reduces neutralization by most of a large panel of potent monoclonal antibodies and antibodies under commercial development (88). The impact of omicron on disease severity, death and hospitalization is yet to be answered.

## Viral Load as a Predictor of COVID-19 Severity

A cumulative body of data has indicated that the viral load is one likely factor of COVID-19 severity, as it is the case of other viral diseases. The relationship between SARS-CoV-2 viral load and risk of disease progression in COVID-19 patients remains undefined. In this section, we summarize the scientific evidence and the major findings from clinical studies, highlighting how viral load could be linked to a disease severity in COVID-19 patients.

In one of the first studies assessing the link between viral load and COVID-19 disease severity, Liu et al. analyzed the viral RNA shedding patterns by RT-qPCR in COVID-19 patients classified with mild and severe disease using samples from 76 patients (8). They found that viral load in nasopharyngeal specimens of severe cases was around 60 times higher than mild cases, and this positive correlation was maintained during the first 12 days of infection (8). In a further study, SARS-CoV-2 RNA viral shedding was evaluated in 3,497 samples (serum, respiratory, stool, and urine) from 96 consecutively admitted patients in a hospital in Zhejiang province, China (15). Viral load in respiratory samples, but not in stool and serum samples, of patients with severe disease was higher than in patients with mild disease. In severe ill patients, male gender and old age were associated with longer duration of virus shedding (15).

In a retrospective cohort study, the predictive power of several reported previously identified prognosis marker [circulating lymphocytes, IL-6, lactic acid, procalcitonin, CRP (C-reactive protein), and viral load] of 142 COVID-19 patients (6) were assessed. In this cohort, non-survivors had higher SARS-CoV-2 load in oropharyngeal swabs when compared to survivors. They authors of the study suggested that circulating lymphocytes, CRP, procalcitonin, IL-6, and viral load could serve as predictors for disease typing and guide classification of COVID-19 patients, and that circulating lymphocytes was the most reliable and sensitive predictor biomarker (6).

We analyzed the viral shedding patterns in nasopharyngeal specimens of 388 Brazilian patients with different forms of COVID-19 (10). Our results revealed that severe patients had higher viral load when compared to patients with mild disease, after 14 days of symptom onset. On the other hand, there was no statistically significant difference in viral load according to severity of COVID-19 in patients at early stage of infection (up to 14 days of symptoms onset) (10).

Pajudas et al. investigated the viral load of SARS-CoV-2 in nasopharyngeal swabs at time of diagnosis in a large cohort (*n* = 1,145) of hospitalized patients and found that survivors showed lower viral loads (*n* = 807; mean log10 viral load 5.2 copies per mL) than non-survivors (*n* = 338; 6.4 copies per mL) (142). Westblade et al. examined the SARS-CoV-2 viral load in 100 patients with cancer and 2,914 without cancer who were admitted to three New York City hospitals. In the overall cohort, the in-hospital mortality rate was 38.8% among patients with a high viral load, 24.1% among patients with a medium viral load, and 15.3% among patients with a low viral load (*p* < 0.001); and importantly, this association was also observed in patients with cancer (143). Fajnzylber et al. quantified SARS-CoV-2 viral load from the respiratory tract, plasma and urine of 231 patients with a diverse range of COVID-19 severity. They concluded that SARS-CoV-2 viral loads, especially in plasma, were associated with increased risk of mortality (144). In Spain, Calle et al. assessed the influence of viral load on the development of respiratory failure during admission in 455 sequential patients. They found that Ct value < 25 in nasopharyngeal samples was associated with increased risk of respiratory failure during admission (OR: 2.99, 95% IC: 1.57–5.69) and suggested that SARS-CoV-2 viral load at time of admission is a valuable predictor for COVID-19 severity (145).

However, the association between SARS-CoV-2 viral load and COVID-19 severity and outcome has not been consistently demonstrated in humans. A study in South Korea did not find any difference in viral load between asymptomatic vs. symptomatic patients (146). This was corroborated by a study in Turkish patients in which the viral load was not a critical factor for hospitalization and mortality among COVID-19 patients (147). The possibility of a type 2 error should be considered. However, it should be noted that not finding a statistical significance does not mean that no difference exists. Another factor that should be taken into account is that most studies that assess the viral load among COVID-19 patients just considered the Ct value for analysis, instead of the number of RNA copies/per milliliter (mL). In fact, Ct values are correlated with the amount of viral RNA in a patient sample (148). However, Ct values cannot be directly compared across RT-qPCR tests and, therefore, they must be interpreted with caution (148). Since many technical issues (differences in protocols, threshold values, viral target, enzymes and research kits, primers, calibration of RT-qPCR machine, period of sample collection, and type of biological specimens) that might impact and alter the Ct value during RT-qPCR reactions, this can represent a bias during the statistical analysis. We suggest that further studies consider using the RNA copies/per mL for viral load analysis among COVID-19 patients. A better comparative standard, combined with the evaluation of host-related factors (e.g., age, sex, comorbidities, etc.) (149) will be crucial to elucidate the real impact of SARS-CoV-2 viral load on COVID-19 disease severity.

## Viral Load and Exposure Dose as a Predictor of COVID-19 Transmission and Severity in Animal Models

Because it would be unethical to assess how viral exposure dose would impact COVID-19 outcome in human controlled experiments, studies using animal models are being used to answer this question. Evidence from animal-based experiments has supported the notion that viral dose could impact disease outcome. The pathogenicity and replication of SARS-CoV-2 in hamsters infected with a low and high viral dose has given clues in that direction. No difference in viral replication titers in several organs were found. However, animals infected with high dose of SARS-CoV-2 had worse outcomes compared to those infected with the low dose (150). Similar results were obtained in mice, in which SARS-CoV-2 resulted in a dose-dependent lethal disease course of infection (151). A recent publication demonstrated that ferrets infected with a high (5 × 10^6^ PFU) and medium (5 × 10^4^ PFU) dose of SARS-CoV-2 had a more consistent upper respiratory tract viral RNA shedding and more severe lung pathology than animals infected with low viral dose (5 × 10^2^ PFU) (152). Intranasal inoculation of ferrets with a high (5 × 10^6^ PFU) or medium (5 × 10^4^ PFU) doses of SARS-CoV-2 resulted in RNA viral shedding in nasal secretions of 100% of the animals; however, only 16.7% of the ferrets in the low challenge dose group (5 × 10^2^ PFU) had detectable viral RNA in the nasal wash. In addition, live SARS-CoV-2 was not detected only in the nasal wash of the medium and low dose groups (152). Overall, a dose-dependent effect was observed in the clinical disease and histopathology (152). In non-human primates, aerosol exposure of cynomolgus macaques (*Macaca fascicularis*) with different doses of SARS-CoV-2 (5–906 TCID_50_) showed that the probability of infection and subsequent disease presentation were dose-dependent. The median infectious dose was 52 TCID_50_ (95% CI: 23–363 TCID_50_) for seroconversion and 256 TCID_50_ (95% CI: 102–603 TCID_50_) for fever development (153). The very low infectious dose of SARS-CoV-2 in this model supports the high transmissibility of the virus in humans.

The relative contribution of different transmission routes (intranasal, aerosol and fomite exposure) was evaluated in hamsters. Intranasal and aerosol exposure resulted in higher viral shedding and more severe disease compared to fomite exposure. Of the three routes studied, aerosol exposure resulted in more rapid virus replication in the lung and weight loss compared to intranasal inoculation and fomite exposure (154). The effects of mask wearing in reducing virus exposure dose and disease severity have been assessed in the hamster model. Hamsters placed in cages separated by a surgical mask partition were shown to be less likely to get infected by SARS-CoV-2 and if they did acquire the illness, it was milder than in animals not protected by a mask. Suggesting that the exposure dose is associated with disease severity (155).

## Association Between Viral Load and Disease Severity in the Context of SARS-CoV-2 Variants

In the context of emerging variants of SARS-CoV-2, recent studies have investigated the impact of SARS-CoV-2 variants on disease severity. In a retrospective study, Ong et al. compared the outcomes of patients infected with alpha, beta, and delta with wild-type SARS-CoV-2 lineages from early 2020 (73). A total of 829 patients infected with these three VOCs in Singapore were enrolled in the study. After adjusting for age and sex, infection by the delta variant was linked with higher odds of oxygen requirement, ICU admission, or death [adjusted odds ratio (aOR), 4.90; 95% confidence interval (CI): 1.43–30.78], while these differences were not seen with alpha and beta variants. Vaccination status was associated with decreased severity. The delta variant was associated with significantly lower Ct values (≤30) and longer viral shedding (median duration 18 days for delta variant, while 13 days for wild type) (73). Taken together, these results suggest that infections with the delta variant feature higher peak viral loads than those in other SARS-CoV-2 variants. This finding also corroborates with outcomes obtained by other research groups, which showed that the delta SARS-CoV-2 variant has a higher viral load than beta and alpha variants in respiratory specimens obtained from COVID-19 patients (56). Within the same perspective, Teyssou et al. compared the relative viral load of the beta variant with alpha variant (156). Using a total of 643 RT-qPCR SARS-CoV-2 positive nasopharyngeal samples, they showed that the beta variant presented an intermediate relative viral load between the alpha other SARS-CoV-2 lineages in nasopharyngeal samples at diagnosis (156).

With regards to the relationship between disease severity and the emerging variants of SARS-CoV-2, a recent meta-analysis study investigated the relationship between SARS-CoV-2 variants and COVID-19 severity (157). Analyzing 26 studies from June 1, 2020, to October 15, 2021, they observed that alpha, beta, gamma, and delta SARS-CoV-2 variants were all more concerning than the wild-type virus in terms of hospitalization, ICU admission, and mortality (157). Interestingly, COVID-19 patients with beta and delta variants have a higher risk to develop severe clinical outcomes even death, when compared to patients with alpha and gamma variants (157).

In South Africa, a recent study investigated the breakthrough infections during periods of circulating beta, delta and omicron VOCs, among healthcare workers participating in the Sisonke phase 3B Ad26.COV2.S vaccine trial (158). Analyzing the data collected between 17 February and 15 December 2021, a total of 40,538 breakthrough infections were observed, resulting in 609 with beta, 22,279 with delta, and 17,650 with omicron. These findings revealed that the omicron variant was associated with a high number of breakthrough infections during the first 30-days of the omicron period in South Africa, while it was linked to less severe disease among COVID-19 patients (158). Interestingly, this finding also corroborate with recent insights achieved about the replication competence of the omicron variant in *ex vivo* explant cultures of human bronchus and lung, suggesting that the lower replication competence of omicron in human lung may be compatible with reduced severity in COVID-19 patients (141). However, further studies are required to confirm this hypothesis since the determinants of severe disease are multifactorial.

## Viral Load in Vaccinated and Unvaccinated Individuals

The primary aim of COVID-19 vaccination is to protect individuals against clinical disease and death, ideally also reducing SARS-CoV-2 transmission in the human population. Although the approved COVID-19 vaccines are safe and effective, they do not provide sterilizing immunity, i.e., viral shedding can occur in vaccinated persons upon exposure to SARS-CoV-2. In addition, breakthrough infections have been reported in vaccinated individuals, although these cases tend to be much milder than in naïve individuals (34, 159, 160). Overall, real world data from several countries have unequivocally shown that vaccine deployment has dramatically reduced the number of COVID-19 associated infections, hospitalizations, and deaths (161, 162).

Some studies have analyzed breakthrough infections mRNA-based (BNT162b2—Pfizer) vaccine recipients, which is being used in many countries around the world. In Israel, its efficiency was 90% in preventing asymptomatic infection, suggesting a potential for halting virus spread (162). Previous studies done in Tel Aviv and Pittsburgh have ratified the role of BNT162b2 (Pfizer) vaccine in the control of the COVID-19 pandemic. In both studies, it was observed a lower viral load in nasal secretions vaccinated compared to non-vaccinated individuals was observed (163, 164). Evaluation of viral shedding after the first dose of the BNT162b2 (Pfizer) vaccine demonstrated that the viral load in nasal secretions was substantially reduced in vaccinated individuals (*n* = 1,888) compared to demographically matched unvaccinated controls (*n* = 1,888) (165).

On the other hand, SARS-CoV-2 shedding did not differ between vaccinated (BNT162b2 vaccine) and non-vaccinated healthcare workers infected with the alpha variant, suggesting potentially reduced efficacy of BNT162b2 in preventing transmission of this variant (166). Similar findings were reported in studies conducted in California (167), Wisconsin (168), Massachusetts (80), and Singapore (169) against the delta variant.

A study done with 3720 Italian healthcare workers fully vaccinated with the BNT162b2 vaccine showed that the 100-day cumulative incidence of vaccine breakthrough infections by the alpha variant was 0.93% in vaccinated vs. 5.78% in non-vaccinated individuals (170). Furthermore, antibody and T-cell responses are not reduced in subjects with breakthrough infection (170). Other studies have been characterized SARS-CoV-2 breakthrough infections in individuals fully vaccinated with mRNA vaccines (BNT162b2 and mRNA-1273). More recently, 14 breakthrough infections have been reported among vaccinated individuals by Deng et al. (159). In that study, half of the cases were immunosuppressed subjects who developed severe disease and required hospitalization (159). Sequencing analysis of infecting virus revealed four distinct SARS-CoV-2 variants, including the alpha and gamma (159). High viral load was detected in symptomatic and asymptomatic patients regardless of disease severity, highlighting the vulnerability of immunosuppressed individuals to post-vaccination infections by diverse variants of SARS-CoV-2 (159).

SARS-CoV-2 viral dynamics were investigated in a prospective, longitudinal study with 173 participants (37 vaccinated and 136 unvaccinated) in the USA using 19,941 patient samples (171). Among them, 36 participants were infected with the alpha variant, 36 participants with the delta variant, and 41 participants with a non-VOC SARS-CoV-2 (171). They found no meaningful difference in the mean peak viral load, shedding duration, clearance duration, or duration of acute infection of either the variants as compared with non VOC (171). Breakthrough infections in vaccinated individuals showed a faster clearance time (5.5 days) compared to non-vaccinated individuals (7.5 days), demonstrating a shorter overall shedding duration among vaccine recipients (171).

A similar prospective and longitudinal study was conducted in the UK in order to investigate the delta variant viral load kinetics in vaccinated [BNT162b2 (Pfizer), AZD1222 (AstraZeneca) and CoronaVac (Sinovac)] and unvaccinated individuals (172). The results revealed that the fully vaccinated individuals with delta variant had a faster mean rate of viral load decline when compared to non-vaccinated individuals with pre-alpha, alpha, or delta variant infections (172). Moreover, it was found that the faster viral load growth was correlated with higher peak viral load and slower decline among individuals (172). In summary, the authors suggested that vaccination reduced the risk of delta variant infection and accelerated viral clearance (172).

A retrospective multicenter cohort study of 17 hospitals in Israel detailed 152 breakthrough infections in fully vaccinated with the BNT162b2 vaccine who developed COVID-19 disease more than 7 days after the second vaccine dose and required hospitalization (173). The cohort was characterized by a large proportion of patients with comorbidities (96%) and immunodepression (40%) (173). They found that higher SARS-CoV-2 load was associated with a significant risk for poor outcome (173). By sequencing analysis, SARS-CoV-2 sequences indicated that most breakthrough infections were caused by the alpha variant (89%) followed by the wild-type virus (7%), and the beta variant (4%) (173).

More recently, another study conducted in Israel analyzed the viral load of 16,000 infections during delta-VOC after vaccination and booster with BNT162b2 vaccine (174). The breakthrough infections in recently fully vaccinated individuals by delta variant showed a lower viral load in comparison to non-vaccinated individuals, but this effect started to decline 2 months after vaccination and vanished 6 months or longer after vaccination (174). In addition, it was found that the effect of the BNT162b2 vaccine on reducing breakthrough infections viral loads is restored after a booster dose (174). Taken together, these findings suggest that BNT162b2 vaccine might decrease the infectiousness of breakthrough infections even with the delta variant (174). However, this protective effect declines over time, but it can be restored with a third vaccine dose (174).

## Final Considerations and Public Health Perspectives

In conclusion, a cumulative body of evidence from animal models and clinical studies has indicated that the SARS-CoV-2 viral load is one likely determinant of ultimate COVID-19 severity and prognosis. The viral exposure dose is also a key determinant to a vaccine protective efficacy (175) and should be kept in mind as vaccine coverage of COVID-19 expands around the world. Despite the current approved COVID-19 vaccines are effective against severe forms of COVID-19, but they are not 100% effective in preventing infection. With is mind, breakthrough infections have been reported in vaccinated individuals, although these cases tend to be much milder than in naïve individuals, especially by the delta variant, which is more transmissible when compared to other SARS-CoV-2 variants. Overall, real world findings from several countries have unequivocally shown that vaccine deployment has dramatically reduced the number of COVID-19 cases, ICU admissions, and deaths. Thus, strategies to reduce viral exposure dose such as masking, frequent hand washing, avoid close contact, mouth and nose covering when coughing, frequently cleaning and disinfecting touched surfaces are crucial to prevent new cases of COVID-19 in the human population. These measures, combined with high coverage vaccination and booster shots, will be crucial to control the COVID-19 and prevent the emergence and spread of new SARS-CoV-2 variants.

In the context of emerging SARS-CoV-2 variants, the recent findings suggest that alpha, beta, gamma, and delta SARS-CoV-2 variants are more serious than the wild-type virus in terms of hospitalization, ICU admission, mortality, and are associated with higher viral loads. Within the SARS-CoV-2 VOCs, patients with beta and delta variants have a higher risk to develop severe clinical outcomes even death, when compared to infected patients with alpha and gamma variants.

## Author Contributions

LP, AK, and SS conceived the work. SS, SL, RS, and LP wrote the original draft. SS, SL, RS, AK, and LP reviewed the final draft. LP supervised the work. All authors contributed to the article and approved the submitted version.

## Funding

The work in LP's lab was funded by the Fiocruz Inova Program and the Foundation for Science and Technology of Pernambuco – FACEPE, Brazil (Grant No. APQ-0560-2.12/19). SS was recipient of a Ph.D. fellowship sponsored by the Foundation for Science and Technology of Pernambuco (FACEPE) Brazil, (Grant No. IBPG-1321-2.12/18). AK was supported by UK Medical Research Council (Grant No. MC_UU_12014/8). The funders had no role in study design, data collection and analysis, decision to publish, or preparation of the manuscript.

## Conflict of Interest

The authors declare that the research was conducted in the absence of any commercial or financial relationships that could be construed as a potential conflict of interest.

## Publisher's Note

All claims expressed in this article are solely those of the authors and do not necessarily represent those of their affiliated organizations, or those of the publisher, the editors and the reviewers. Any product that may be evaluated in this article, or claim that may be made by its manufacturer, is not guaranteed or endorsed by the publisher.
